# Label-set impact on deep learning-based prostate segmentation on MRI

**DOI:** 10.1186/s13244-023-01502-w

**Published:** 2023-09-25

**Authors:** Jakob Meglič, Mohammed R. S. Sunoqrot, Tone Frost Bathen, Mattijs Elschot

**Affiliations:** 1 Department of Circulation and Medical Imaging, Norwegian University of Science and Technology - NTNU, 7030 Trondheim, Norway; 2 Faculty of Medicine, University of Ljubljana, 1000 Ljubljana, Slovenia; 3 Department of Radiology and Nuclear Medicine, St. Olavs Hospital, Trondheim University Hospital, 7030 Trondheim, Norway

**Keywords:** Label, Deep learning, Segmentation, Prostate, MRI

## Abstract

**Background:**

Prostate segmentation is an essential step in computer-aided detection and diagnosis systems for prostate cancer. Deep learning (DL)-based methods provide good performance for prostate gland and zones segmentation, but little is known about the impact of manual segmentation (that is, label) selection on their performance. In this work, we investigated these effects by obtaining two different expert label-sets for the PROSTATEx I challenge training dataset (*n* = 198) and using them, in addition to an in-house dataset (*n* = 233), to assess the effect on segmentation performance. The automatic segmentation method we used was nnU-Net.

**Results:**

The selection of training/testing label-set had a significant (*p* < 0.001) impact on model performance. Furthermore, it was found that model performance was significantly (*p* < 0.001) higher when the model was trained and tested with the same label-set. Moreover, the results showed that agreement between automatic segmentations was significantly (*p* < 0.0001) higher than agreement between manual segmentations and that the models were able to outperform the human label-sets used to train them.

**Conclusions:**

We investigated the impact of label-set selection on the performance of a DL-based prostate segmentation model. We found that the use of different sets of manual prostate gland and zone segmentations has a measurable impact on model performance. Nevertheless, DL-based segmentation appeared to have a greater inter-reader agreement than manual segmentation. More thought should be given to the label-set, with a focus on multicenter manual segmentation and agreement on common procedures.

**Critical relevance statement:**

Label-set selection significantly impacts the performance of a deep learning-based prostate segmentation model. Models using different label-set showed higher agreement than manual segmentations.

**Key points:**

• Label-set selection has a significant impact on the performance of automatic segmentation models.

• Deep learning-based models demonstrated true learning rather than simply mimicking the label-set.

• Automatic segmentation appears to have a greater inter-reader agreement than manual segmentation.

**Graphical Abstract:**

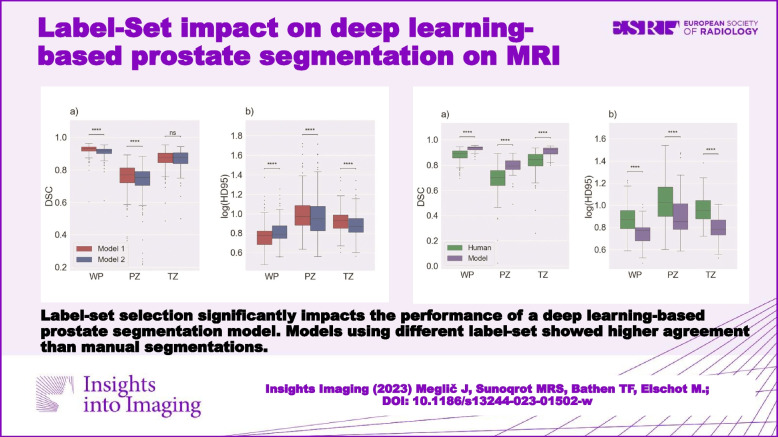

**Supplementary Information:**

The online version contains supplementary material available at 10.1186/s13244-023-01502-w.

## Introduction

Prostate cancer (PCa) is a major health concern, ranking as the fifth leading cause of cancer-related deaths in men, and the second most common cancer among men worldwide [1]. Accurate diagnosis and early detection of PCa is essential for effective treatment of this disease [2]. Multiparametric magnetic resonance imaging (mpMRI) has been internationally established as a valuable diagnostic tool for PCa [3]. Currently, radiologists manually interpret mpMRI images. However, this approach has a number of limitations, including inter-reader variability. Automated computer-aided detection and diagnosis systems (CAD), can help overcome these limitations [4–6].

Region-of-interest (ROI) segmentation is a crucial step in CAD systems for PCa [4]. Deep learning (DL)-based methods have shown promising results in terms of performance. In several studies, convolutional neural network (CNN)-based methods have been shown to achieve high accuracy, with some achieving segmentation performance that is comparable to expert radiologists [7].

In recent years, several CAD systems have been developed for PCa, and promising results have been reported in the literature [8, 9]. In addition, several commercial prostate gland and zone segmentation products are currently available [6]. In clinical practice, these segmentations are used to estimate the gland volume for calculation of the prostate-specific antigen (PSA) density, and for real-time fusion during targeted ultrasound-guided prostate biopsies [6]. However, most of these models have been trained with low variability in datasets, mainly images and manual, ground-truth, segmentations (that is, labels) from a single institution.

Manual segmentation of the prostate gland is a time-consuming and difficult task that is subject to inter-reader variability [10]. This variability can arise from differences in the training and experience of the radiologists, as well as variations in the imaging equipment and protocols used. Despite this, manual segmentation is still considered as the gold standard in prostate gland segmentation. Nevertheless, many of the published segmentation methods refer to the need for a larger, multi-institutional image dataset to improve segmentation performance [8, 9]. However, the impact of label-set selection on the performance of these models has not been investigated.

It can be considered common knowledge that the accuracy and reliability of DL-based segmentation models rely on the quality of labels. Therefore, it is crucial to consider — and quantify — the impact of label selection on the performance of DL-based prostate segmentation models. This will help to improve the accuracy and reliability of CAD systems for PCa and ultimately lead to better diagnosis and treatment of this disease.

In this study, we aimed to investigate and quantify the impact of label selection for training and evaluation on the performance of DL-based prostate segmentation models.

## Methods

### Datasets

In this study, we used transverse T2-weighted (T2W) MR images from two datasets: the publicly available PROSTATEx I training dataset (*n* = 204; 6 cases were excluded due to segmentations mismatching) [11], and an in-house collected multiparametric 3 T MRI dataset (*n* = 233).

The in-house collected dataset was obtained from St. Olavs Hospital, Trondheim University Hospital, Trondheim, Norway between March 2015 and December 2017 as part of a previous prospective study [12]. It consists of mpMR images from 233 patients (median age = 65; range: 44–76 years) who were examined due to suspicion of prostate cancer, via the Norwegian standardized care pathway, in which patients with elevated PSA and/or abnormal digital rectal exam results are referred for an initial mpMRI scan to identify suspicious cancerous tissue. T2W imaging was performed on a Magnetom Skyra 3 T MRI system (Siemens Healthineers, Erlangen, Germany) with a turbo spin-echo sequence (repetition time/echo time = 4450–9520/101–108 ms, 320 × 320–384 × 384 matrix size, 26–64 slices, 3–3.5 mm slice thickness and 0.5 × 0.5–0.6 × 0.6 mm^2^ in-plane resolution).

### Manual segmentation

Manual segmentation of the peripheral zone (PZ) and transition zone (TZ) of the prostate for the in-house collected dataset was performed using ITK-SNAP (version 3.6.0) [13] by a radiology resident at St. Olavs Hospital, Trondheim University Hospital, Trondheim, Norway, who was trained by a radiologist with more than 10 years of experience in prostate imaging. Segmentation of the whole prostate (WP) resulted from the union of PZ and TZ. Lesion segmentation was beyond the scope of this study and was therefore not considered.

For the PROSTATEx dataset, there were two sets of expert manual segmentations (label-set):

- Set A: where segmentation was performed by two radiology residents and reviewed by two expert radiologists. Each pair of resident and radiologist reviewed half of the cases. This label-set was made publicly available by Cucolo, et al. [14].
- Set B: where segmentation was performed by imaging experts with a combined experience of more than 25 years in prostate imaging and reviewed by radiation oncologists at Miller School of Medicine, Miami, USA.

The labels of set A and set B were checked for errors (that is, floating, mis-segmented pixels) and corrected accordingly. For this purpose, a customized method (https://github.com/meglaficus/ProstateSeg_QC) was used to look for the most common manual segmentation errors and highlight the erroneous masks.

### Automatic segmentation

Automatic segmentation of PZ and TZ was performed using a full-resolution 3D nnU-Net model [15] with a fivefold averaging strategy in which the training set is divided into 5-folds, with each fold used to train and validate a submodel. To ensure model comparability, these folds were kept equal for each model used in the same experiment. Each of the 5 submodels is then used to predict case segmentation during testing, and the five predictions are then averaged to create a mask. nnU-Net (version 1.7.0) was trained for 300 epochs and implemented with PyTorch (version 1.11.0) [16] using Python (version 3.9.12; Python Software Foundation, Wilmington, DE, USA) on a single NVIDIA GeForce RTX 2070 Super GPU with 8 GB VRAM. nnU-Net is a self-configuring framework that automatically optimizes preprocessing, model configuration, and training, and thus we did not specify specific training parameters.

In this study, 5 models were developed:

- Model 1: trained with PROSTATEx images, along with their corresponding labels from set A.
- Model 2: trained with PROSTATEx images, along with their corresponding labels from set B.
- Model 3: trained with the in-house images, along with their corresponding labels.
- Model 4: trained with subset of 148 randomly selected patients (to create a 75%/25% training/test split) from PROSTATEx images, along with their corresponding labels from set A.
- Model 5: trained with subset of 148 randomly selected patients from PROSTATEx images, along with their corresponding labels from Set B. Here the same training cases were used as for Model 4.

The masks derived by these models were post-processed by keeping only the central dominant largest 3D connected component, using a pixel connectivity of 26. WP segmentations resulted from the union of PZ and TZ.

### Experiments

1. *Agreement between manually-derived label-sets*
  The first step was to measure the agreement between labels of set A and set B to establish a baseline for comparing DL-based models’ performance and assess agreement between radiologists.
2. *The impact of training label-set selection*
  We compared the masks generated by model 1 (trained on set A) and model 2 (trained on set B), using the in-house dataset as a test set to evaluate the impact of training label-set selection on DL-based model performance.
3. *The impact of testing label-set selection*
  To complement the previous experiment, we compared the masks generated by model 3 (trained on the in-house dataset) for PROSTATEx images with labels from set A and set B, separately, to evaluate the impact of the testing label-set selection on DL-based model apparent performance.
4. *Agreement between algorithm and human segmentations*
  In this experiment, the PROSTATEx dataset was partitioned into a training subset (148 cases; 75%) and a testing subset (50 cases; 25%). Using models 4 and 5, and test label-sets A and B, we evaluated several factors. Firstly, we evaluated the impact of using labels from the same label-set for training and testing, as opposed to using labels from different label-sets for training and testing. Secondly, we compared the performance of Models 4 and 5 with the agreement between both test label-sets (that is, human performance). Thirdly, we compared the agreement between the masks generated by Model 4 and model 5 to the agreement between the two test label-sets.

### Statistical analysis

The Dice similarity coefficient (DSC) [17] and the 95th percentile of the Hausdorff distance (HD95) [18] between two masks were calculated as metrics for segmentation performance and mask agreement. In addition, the relative volume difference (RVD) was calculated as a quantitative measure to analyze and compare the volumes of the segmentations [19]. RVD was the only metric that was applied solely on WP as this is most relevant in a clinical setting.

The Shapiro–Wilk test was used to test the normality of the data sets, and because of the non-Gaussian distribution found, the Wilcoxon signed-rank test, followed by the Bonferroni correction for multiple testing, was used to assess differences in DSC, DH95 and RVD. Corrected *p* values of less than 0.05 were considered statistically significant.

All statistical analysis was performed in Python (version 3.9.12).

## Results

**Fig. 1:**
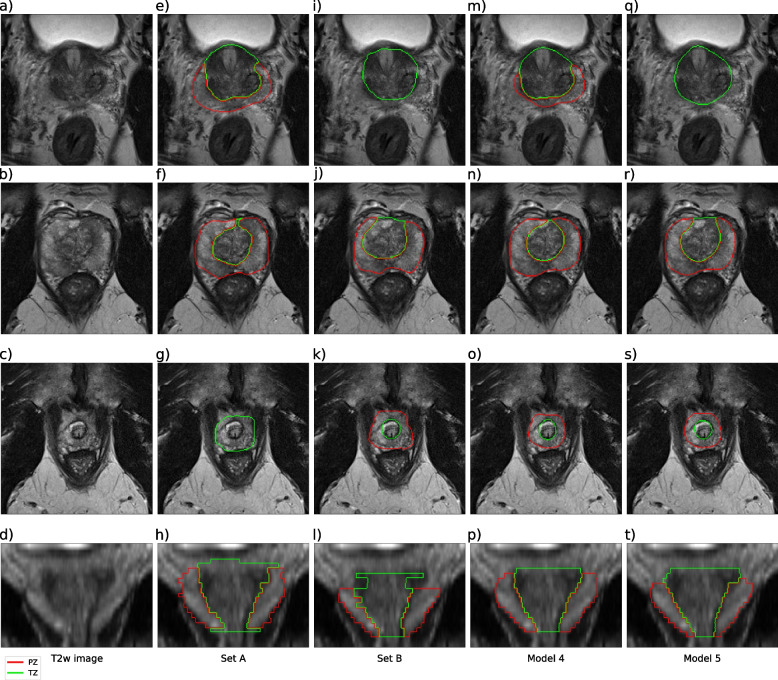
Examples of manual and automatic segmentation. The figure shows segmentations of the transition zone (TZ) and peripheral zone (PZ) of a case from PROSTATEx that is part of the test set for models 4 and 5. Axial images of the prostate base, mid, and apex sections are shown in rows 1, 2, and 3, respectively. A coronal image of the prostate is shown in row 4. **a**–**d** T2-weighted images, the rest are segmentations from set A (**e**–**h**), set B (**i**–**l**), model 4 (**m**–**p**), and model 5 (**q**–**t**)

Training of all models had converged after 300 epochs. The loss function plots are provided as supplementary material, shown in Fig. S1. Examples of human and model segmentations for one case are shown in Fig. 1.

### Agreement between manually derived label-sets

Comparison of the agreement between the manually derived set A and set B yielded a median DSC of 0.891, 0.703, and 0.847, and a median HD95 of 7.41, 10.55, and 9.00 mm for WP, PZ, and TZ, respectively. The median RVD was 9.27% for WP, using set B as reference.

### The impact of training label-set selection

**Fig. 2:**
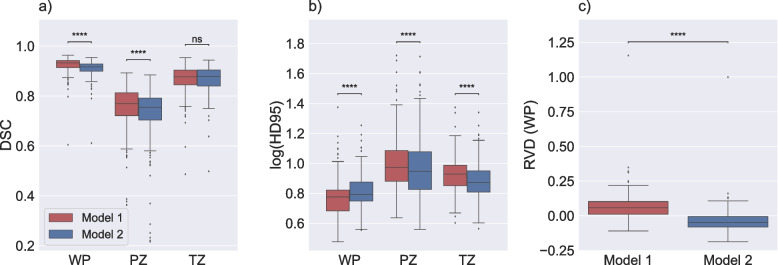
Performance of model 1 and model 2. Boxplots displaying the Dice similarity coefficient (DSC) (**a**), the 95th percentile of the Hausdorff distance (HD95; log applied to improve visualization) (**b**), and the relative volume difference for whole prostate (RVD (WP)) (**c**) comparing the performance of model 1, which is trained with set A and model 2, which is trained with set B on the same test set (in-house). The median DSC and HD95 for whole prostate (WP), peripheral zone (PZ), and transition zone (TZ) were 0.933, 0.769, and 0.877 and 5.97, 9.41, and 9.50 mm, respectively, for model 1. The median DSC and HD95 for WP, PZ, and TZ were 0.916, 0.754, and 0.878 and 6.18, 8.88, and 7.44 mm, respectively, for model 2. The median RVD (WP) were 6.66% and − 3.73% for model 1 and model 2, respectively. ns: *p* ≥ 0.05, **** *p* < 0.0001

The comparison of model 1 and model 2 performance is illustrated in Fig. 2. The results indicated that the selection of the training label-set had a significant impact on model performance in an independent test set, with model 1 (set A) scoring higher on DSC and HD95 than model 2 (set B) for whole-prostate segmentation in the in-house collected dataset. In addition, there was a significant difference in RDV between the models. Model 1 demonstrates a tendency to over-segment the prostate (with a median RVD of 6.66%), while model 2 tends to under-segment the prostate (with a median RVD of − 3.73%).

### The impact of testing label-set selection

**Fig. 3:**
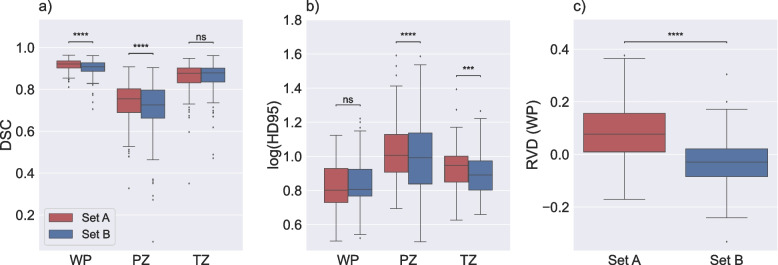
Performance of model 3 on set A and set B**.** Boxplots displaying the Dice similarity coefficient (DSC) (**a**), the 95th percentile of the Hausdorff distance (HD95; log applied to improve visualization) (**b**), and the relative volume difference for whole prostate (RVD (WP)) (**c**) comparing agreement between the masks of the PROSTATEx dataset generated by model 3 with set A and set B, separately. The median DSC and HD95 for whole prostate (WP), peripheral zone (PZ), and transition zone (TZ) were 0.922, 0.755, and 0.878 and 6.34, 10.16, and 8.86 mm, respectively, compared with set A. The medians DSC and HD95 for WP, PZ, and TZ were 0.908, 0.726, and 0.879 and 6.40, 9.80, and 7.78 mm, respectively, compared with Set B. The median RVD (WP) were 8.52% and − 3.19% for model 1 and model 2, respectively. ns: *p* ≥ 0.05, *** *p* < 0.001, **** *p* < 0.0001

The comparison of generated masks by model 3 with set A and set B as a reference is illustrated in Fig. 3. The results indicated that the selection of the independent testing label-set had a significant impact on apparent model performance for WP and PZ with DSC, and PZ and TZ with HD95. In addition, there was a significant difference in RDV between the label-sets. The results resemble those of the prior experiment, indicating a clear inclination towards over-segmentation when evaluating against set A (with a median RVD of 8.52%), as well as an inclination towards under-segmenting the prostate when evaluating against Set B (with a median RVD of − 3.19%).

### Agreement between algorithm and human segmentations

**Fig. 4:**
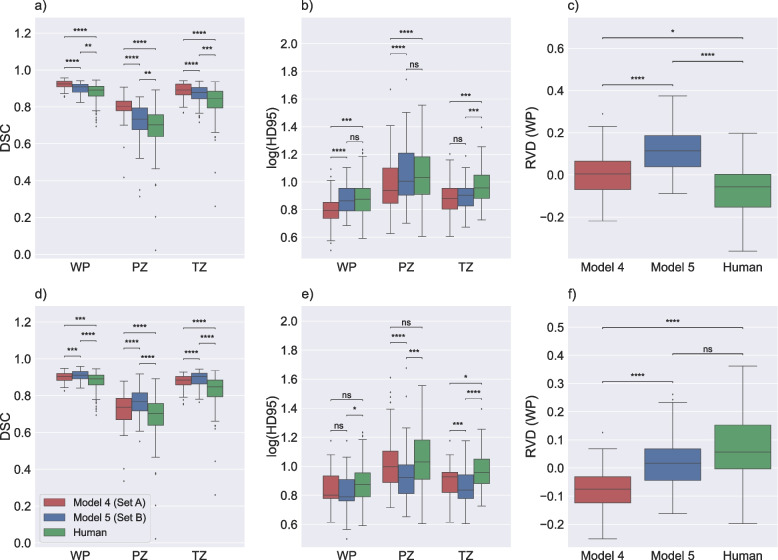
Performance of model 4, model 5 and manual segmentations on set A and set B. Boxplots displaying the Dice similarity coefficient (DSC) (**a** and **d**), the 95th percentile of the Hausdorff distance (HD95; log applied to improve visualization) (**b** and **e**), and relative volume difference for whole prostate (RVD (WP)) (**c** and **f**) comparing agreement between the masks of the test subset of the PROSTATEx dataset generated by model 4 and model 5 with set A (**a**–**c**), and set B (**d**–**f**) as reference, in addition to the similarity between set A and set B (human). ns: *p* ≥ 0.05, * *p* < 0.05, ** *p* < 0.01, *** *p* < 0.001, **** *p* < 0.0001

The comparison of generated masks by model 4 and model 5 with labels from set A and set B is shown in Fig. 4 and Table 1. As expected, the results indicated that when the masks are compared to the same label-set used for model training, the agreement (for DSC and HD95) was significantly higher than when a different label-set was used for training. Similarly, the median RVD was closer to 0 when the training set and testing set came from the same label-set. However, interestingly, the agreement between the human segmentations of set A and set B for the test subset was significantly lower (for DSC and most of 95HD) than the agreement between the masks generated by a model trained with one label-set and tested on another. Furthermore, the variability in RVD of the models was smaller than that of the human segmentations.

**Table 1:**
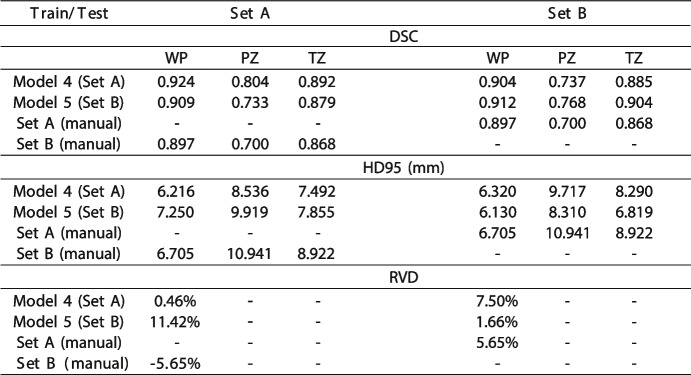
Performance of model 4, model 5, and similarity between manual segmentations

Median Dice similarity coefficient (*DSC*), the 95.^th^ percentile of the Hausdorff distance (*HD95*; in mm), and relative volume difference (*RVD*) displaying the performance of model 4 and model 5 with set A and set B as reference, in addition to the similarity between set A and set B (human)

**Fig. 5:**
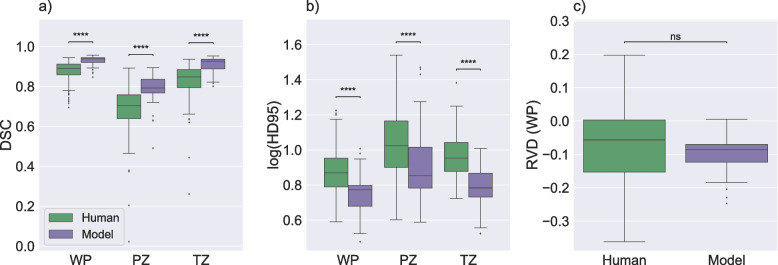
Agreement between generated segmentations and manual segmentations. Boxplots displaying the Dice similarity coefficient (DSC) (**a**), the 95th percentile of the Hausdorff distance (HD95; log applied to improve visualization) (**b**), and relative volume difference for whole prostate (RVD (WP)) (**c**). Panels **a** and **b** compare scores that measured agreement between generated masks derived from model 4 and model 5 and scores that measured the agreement between the manual masks in the test subset of the PROSTATEx dataset. Panel **c** illustrates RVD scores for the same comparisons. ns: *p* ≥ 0.05, **** *p* < 0.0001

The comparison of agreement between generated masks by model 4 and model 5 and agreement between labels from set A and set B in the test subset is shown in Fig. 5. The results showed that the agreement between the automatic segmentations was significantly higher than that of the human experts who segmented their training sets. In addition, it was evident that there was wider variability in RVD between human annotators than between the model-derived segmentation. These results indicated that DL-based segmentation models truly learned to segment the images rather than simply mimicking the training label-set.

## Discussion

Prostate segmentations are utilized in clinical settings to estimate the gland volume, which is necessary for calculating the prostate-specific antigen (PSA) density, and to facilitate real-time fusion during targeted ultrasound-guided prostate biopsies [6]. In addition, ROI segmentation is an essential step in CAD systems for PCa detection [4], and DL-based models have shown to be promising for prostate segmentation [7]. However, the impact of using different label-sets on these models’ performance has not been widely studied. Therefore, this study investigated the impact of label-set selection on the performance of DL-based prostate gland and zone segmentation models. Two different label-sets for the PROSTATEx I challenge dataset were obtained and used, along with an in-house dataset, to train and test the 3D nnU-Net model in five different scenarios. The change in performance as a result of label-set selection was observed and quantified.

The agreement between the two manually derived label-sets found in our experiments was consistent with the inter-observer agreement reported in previous studies [10, 20]. Using these values as a benchmark, the nnU-Net models in the study performed better than the manually derived label-sets, except for HD95 score of TZ in model 1, which was slightly higher, but comparable to that of the manual label-sets. The performance of the nnU-Net models was also comparable to other similar models trained with different training and/or testing sets [21, 22].

Our experiments showed that the selection of both training and testing label-sets has a significant impact on model performance. This implies that model performance should always be evaluated in the context of the ground truth for training and testing and that models trained and/or tested with different label-sets cannot be directly compared. However, one possible solution to overcome the evaluation issue is to benchmark against a public dataset or to participate in open challenges, where all models use the same dataset and label-set to compare their performance [6].

Additionally, methods such as the simultaneous truth and performance level estimation (STAPLE) algorithm have been developed to address the challenges associated with characterizing the performance of image segmentation approaches [23]. It provides a means to estimate the true segmentation from multiple annotators and account for the performance levels of each segmentation. This might be of use for compiling a robust segmentation label-set, when segmentations from three or more annotators are available.

We conducted a comparison of segmented prostate volumes using RVD to assess the impact of label-set choice on gland volumes derived by models trained on those label-sets, revealing significant differences. These findings highlight that the influence of the label-set extends beyond commonly utilized research metrics and has tangible consequences in real-world clinical applications where prostate segmentations are employed for the calculation of the gland volume.

Based on our results, it cannot be stated that a single label-set had superior performance. The results of the second and third experiments indicate that set A generally yields higher DSC scores, but also higher HD95 scores compared to set B. However, this variation in label-sets may not be detrimental and could actually be beneficial in training more generalized segmentation models with multiple label-sets so they can adapt to different variations.

Our analysis of agreement between algorithm and human segmentations supports the conclusion that label selection has a significant effect on model performance. As expected, the results showed that when the segmentation masks are tested with the same label-set used in their model training, the performance is better than when it is tested with labels from different annotators [24, 25]. This is a clear indication that the models are able to learn and adapt to the specific characteristics of the label-set used during training.

At the same time, an interesting finding is that the agreement between the two manually derived label-sets (set A and set B) for the PROSTATEx test subset was significantly lower than the agreement between the masks generated by a model trained with one label-set and tested on another. In addition, we observed wider variation in RVD for manually derived label-sets in comparison with model derived label-sets. This suggests that the models are able to generalize and learn from the images and are relatively robust to variations in label-set. It is indicative of true learning as opposed to simply mimicking the training label-set, which is a significant advancement in the field of medical image segmentation. This finding highlights the potential for these models to be used in real-world scenarios where variations in label-sets are likely to occur. It also opens up opportunities for future research to explore the factors that contribute to the generalization capability of DL-based segmentation models and how this can be further improved.

This study has some limitations that should be considered when interpreting its results. The use of a single DL-based segmentation model architecture (nnU-Net) means that the conclusions may not be applicable to other architectures. However, since nnU-Net is based on the commonly used U-Net architecture, it is likely that similar results would be obtained with other U-Net models [26–28]. Additionally, only two evaluation metrics (DSC and HD95) were used, which may not provide a comprehensive understanding of the segmentation performance of the model. However, these metrics are widely used for prostate cancer segmentation and provide different perspectives on the results, and their findings were confirmed by whole gland volume analysis. Additionally, the study did not analyze the prostate regions (apex, mid, and base) separately, which could have added complexity to the analysis. Despite these limitations, the study provides important insights into the performance of DL-based segmentation models when trained and tested on different label-sets and emphasizes the importance of considering generalization in the development of these models.

## Conclusions

In this study, we investigated the impact of label-set selection on the performance of a DL-based prostate segmentation model. We found that the use of different label-sets of prostate gland and zone segmentations has a measurable impact on model performance. More thought should be given to the label-set, with a focus on multicenter manual segmentation and agreement on common procedures. Furthermore, we found that the predictions made by automatic segmentation models were more consistent than the manually derived segmentations they were trained on. Moreover, DL-based models demonstrated the ability to truly learn from the images, rather than simply mimic the training label-set. This sheds light on their future potential to improve prostate segmentation and standardize decision-making in clinical practice.

### Supplementary Information

**Additional file 1: Figure S1.** Loss function plots for all 5 models.

## Data Availability

• PROSTATEx I challenge training dataset is available at the Cancer Imaging Archive: https://wiki.cancerimagingarchive.net/pages/viewpage.action?pageId=23691656. • Manual segmentation set A is available in the PROSTATEx masks repository: https://github.com/rcuocolo/PROSTATEx_masks. • Sharing of the other datasets used and/or analyzed during the current study will be considered by the corresponding author upon reasonable request.

## Abbreviations

CAD: Automated computer systems for detection and diagnosis
CNN: Convolutional neural network
DL: Deep learning
DSC: Dice similarity coefficient
HD95: 95Th percentile of the Hausdorff distance
mpMRI: Multiparametric magnetic resonance imaging
PCa: Prostate cancer
PSA: Prostate-specific antigen
PZ: Peripheral zone
ROI: Region of interest
RVD: Relative volume difference
STAPLE: Simultaneous truth and performance level estimation
T2W: T2-weighted
TZ: Transition zone
WP: Whole prostate
