# Supplementary material for: Label-set impact on deep learning-based prostate segmentation on MRI
Source: Insights Imaging. 2023 Sep 25;14:157. doi: 10.1186/s13244-023-01502-w (PMC10519913; doi:10.1186/s13244-023-01502-w)

**Label-Set Impact on Deep Learning-Based Prostate Segmentation on MRI**

**ELECTRONIC SUPPLEMENTARY MATERIAL**

For the PROSTATEx dataset, there were two sets of expert manual segmentations (label-set):

- Set A: where segmentation was performed by two radiology residents and reviewed by two expert radiologists. Each pair of resident and radiologist reviewed half of the cases. This label-set was made publicly available by Cucolo, et al.
- Set B: where segmentation was performed by imaging experts with a combined experience of more than 25 years in prostate imaging and reviewed by radiation oncologists at Miller School of Medicine, Miami, USA.

Automatic segmentation of PZ and TZ was performed using a full-resolution 3D nnU-Net model with a 5-fold averaging strategy in which the training set is divided into 5 folds, with each fold used to train and validate a submodel. To ensure model comparability, these folds were kept equal for each model used in the same experiment. Each of the 5 submodels is then used to predict case segmentation during testing, and the five predictions are then averaged to create a mask. nnU-Net (version 1.7.0) was trained for 300 epochs and implemented with PyTorch (version 1.11.0) using Python (version 3.9.12; Python Software Foundation, Wilmington, DE, USA) on a single NVIDIA GeForce RTX 2070 Super GPU with 8 GB VRAM. nnU-Net is a self-configuring framework that automatically optimizes preprocessing, model configuration, and training, and thus we did not specify specific training parameters.

In this study, 5 models were developed:

- Model 1: trained with PROSTATEx images, along with their corresponding labels from Set A.
- Model 2: trained with PROSTATEx images, along with their corresponding labels from Set B.
- Model 3: trained with the in-house images, along with their corresponding labels.
- Model 4: trained with subset of 148 randomly selected patients (to create a 75%/25% training/test split) from PROSTATEx images, along with their corresponding labels from Set A.
- Model 5: trained with subset of 148 randomly selected patients from PROSTATEx images, along with their corresponding labels from Set B. Here the same training cases were used as for Model 4.

Training of all models converged after 300 epochs. The loss function plots are provided in Figure S1.

**Figure S1:** Loss function plots for all 5 models


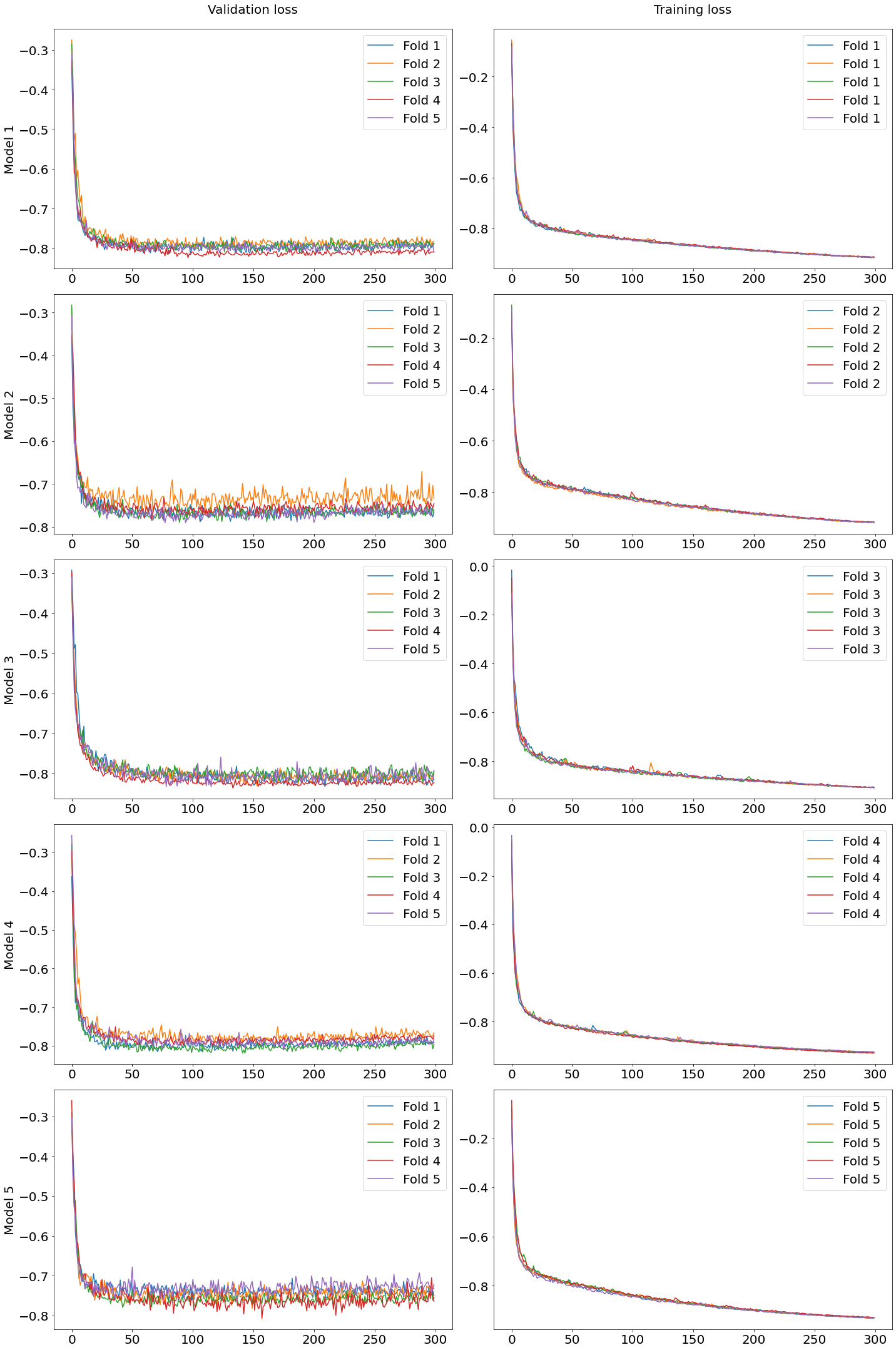

Supplement: Supplementary file 1 — Additional file 1: Figure S1. Loss function plots for all 5 models. [file 13244_2023_1502_MOESM1_ESM.docx]
